# Heterotopic Ossification Following Pediatric Elbow Dislocation: A Case Report

**DOI:** 10.1055/s-0041-1739403

**Published:** 2021-12-16

**Authors:** Henrique Mansur, Roberto Luiz Bisol, Daniel Augusto Maranho

**Affiliations:** 1 Departamento de Ortopedia e Traumatologia, Hospital Santa Helena – Rede D'or e Hospital Regional do Gama, Distrito Federal, Brasil; 2 Departamento de Ortopedia e Traumatologia, Rede Santa, Brasília, DF, Brasil; 3 Departamento de Ortopedia e Traumatologia, Hospital Sírio-Libanês, Brasília, DF, Brasil

**Keywords:** adolescent, child, elbow joint, heterotopic ossification, joint dislocations

## Abstract

Elbow dislocations are relatively uncommon in children, and most cases present with associated fractures. Complete elbow dislocations are relatively rare, and may involve an increased likelihood of severe soft-tissue injuries. A potential complication is the development of heterotopic ossification, which is usually asymptomatic, but may disturb the joint motion. We describe a case of an 11-year-old girl who sustained an elbow dislocation without associated fractures, but with partial distal disruption of the brachialis muscle. Following closed reduction, the patient developed heterotopic ossification in the anterior distal third of the humerus and loss of range of motion. Surgical treatment with excision of heterotopic ossification through a direct lateral approach provided an excellent result. Heterotopic ossification is a potential complication following elbow dislocation in children, and surgical excision through a lateral approach is an option of treatment when there is functional limitation. Before making the decision to perform surgery, the maturation of the ossification process must be observed.

## Introduction

The elbow is the most common major joint to present traumatic dislocation in childhood, although the lesion is relatively rare, representing from 3% to 25% of all pediatric elbow injuries. 1 Pediatric elbow dislocations are complex lesions, associated with fractures in 75% of cases, while simple dislocations (purely ligamentous) in children are associated with a varying degree of soft-tissue injuries. 1 2

Following elbow dislocation, patients might present a varying loss of range of motion (ROM) associated or not with heterotopic ossification (HO), 3 which is the development of mature lamellar bone within tissues beyond the periosteum, such as skeletal muscle, fibrous and capsuloligamentar components, and subcutaneous tissue. 4 In most cases, HO is asymptomatic and detected as an incidental imaging finding. However, it might be painful and associated with focal dysesthesia, signs of inflammation, and decreased ROM. Symptomatic HO around the elbow in children is an uncommon finding, especially without an associated fracture or history of surgery. 4 5 Here, we describe a case of an 11-year-old girl with HO following a traumatic elbow dislocation without associated fractures, but with partial disruption of the brachialis muscle. During the follow-up, the patient experienced persistent loss of flexion, and underwent surgical excision of the HO, followed by normalization of elbow motion and no recurrence.

## Case Report

The present study was approved by the Institutional Review Board (under protocol n° 36203120.6.0000.0023), with signed consent from the participant and her parents.

**Fig. 1:**
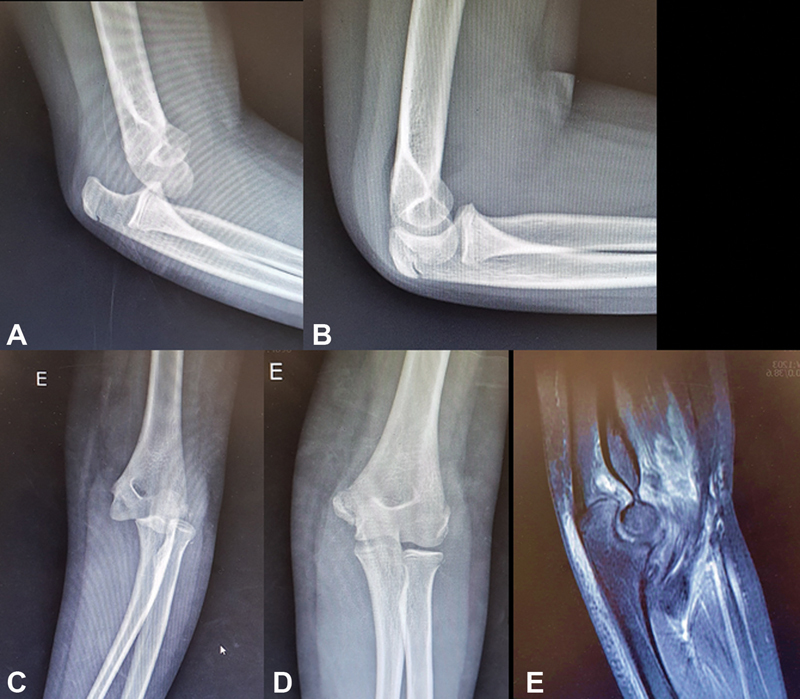
( **A** ) Lateral and ( **B** ) anteroposterior (AP) radiographs of an 11-year-old girl showing posterolateral dislocation of the elbow without concomitant fractures; lateral ( **C** ) and ( **D** ) AP radiographs after closed reduction, showing congruent joint without fractures. ( **E** ) Magnetic resonance imaging (MRI): T2-weighted sagittal scan of the left elbow following the traumatic elbow dislocation after closed reduction. The MRI shows partial rupture of the fibers of the brachialis muscle, and a poorly-defined blood collection of 3.1 cm × 1.8 cm.

**Fig. 2:**
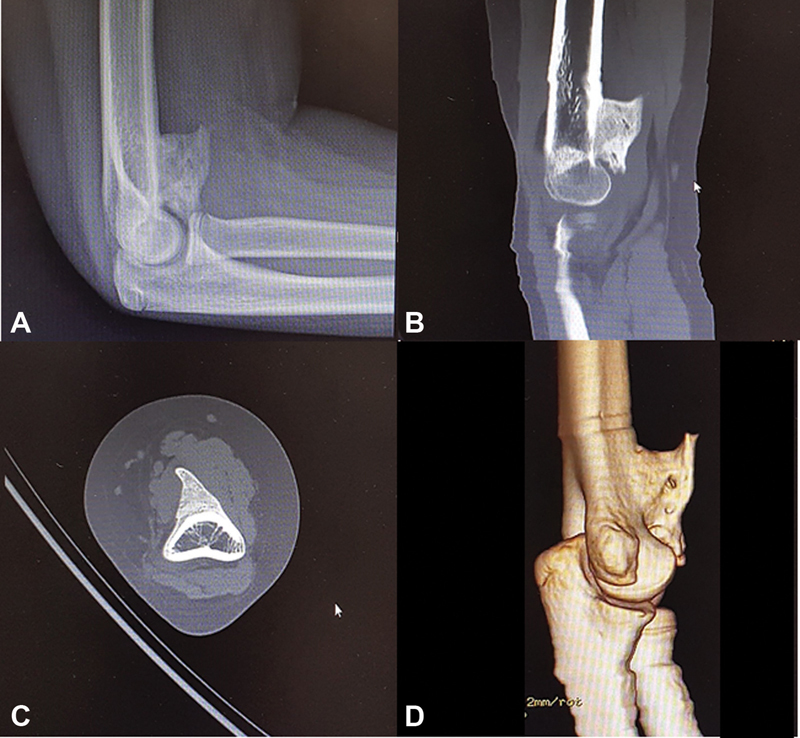
Lateral radiograph ( **A** ) and sagittal ( **B** ), axial ( **C** ) **,** and 3D ( **D** ) computed tomography (CT) scans of the elbow showi g heterotopic ossification (HO) at the anterior aspect of the distal humerus causing potential joint impingement.

An 11-year-old girl was admitted to the emergency room reporting a fall from a small height and indirect trauma to the left elbow. There was no history of musculoskeletal lesion on the affected upper arm. During the physical exam, she presented severe pain, swelling, tenderness, and elbow deformity without neurovascular deficit. The radiographs showed posterolateral dislocation of the elbow without concomitant fractures. A closed reduction under anesthesia was performed, and the elbow was immobilized with an above-elbow plaster splint in 90° of flexion and neutral rotation for one week, followed by two weeks of a shoulder sling. However, because of important elbow edema and ecchymosis, a magnetic resonance imaging (MRI) scan was performed, showing partial rupture of the fibers of the brachialis muscle, and a poorly-defined blood collection ( Fig. 1 ). No specific treatment was performed to address the brachialis muscle lesion. After six months of physical therapy, the patient had a pain-free ROM of ∼ 90° (flexion of 95° and extension deficit of 5°). No loss of pronation or supination was noticed. A sizable solid mass was palpable anteriorly at the distal extremity of the humerus. Radiographs and computed tomography (CT) scans showed HO at the anterior aspect of the distal humerus causing potential joint impingement ( Fig. 2 ). Because of the residual loss of ROM, the decision was to resect the HO surgically.

**Fig. 3:**
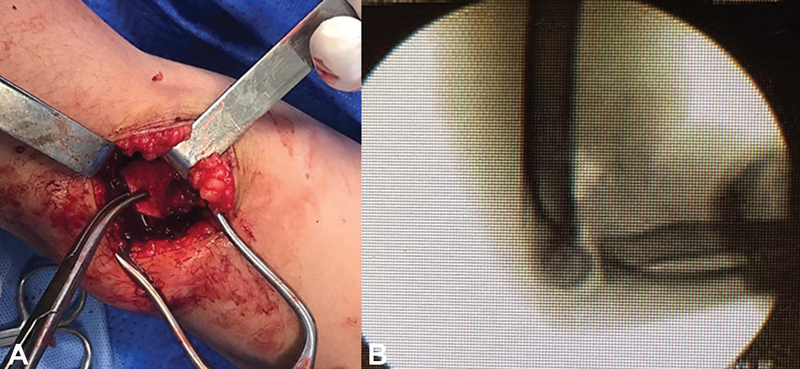
Intraoperative photograph showing HO of the distal humerus through the direct lateral approach ( **A** ). Intraoperative lateral X-ray of the elbow after excision of the HO ( **B** ).

**Fig. 4:**
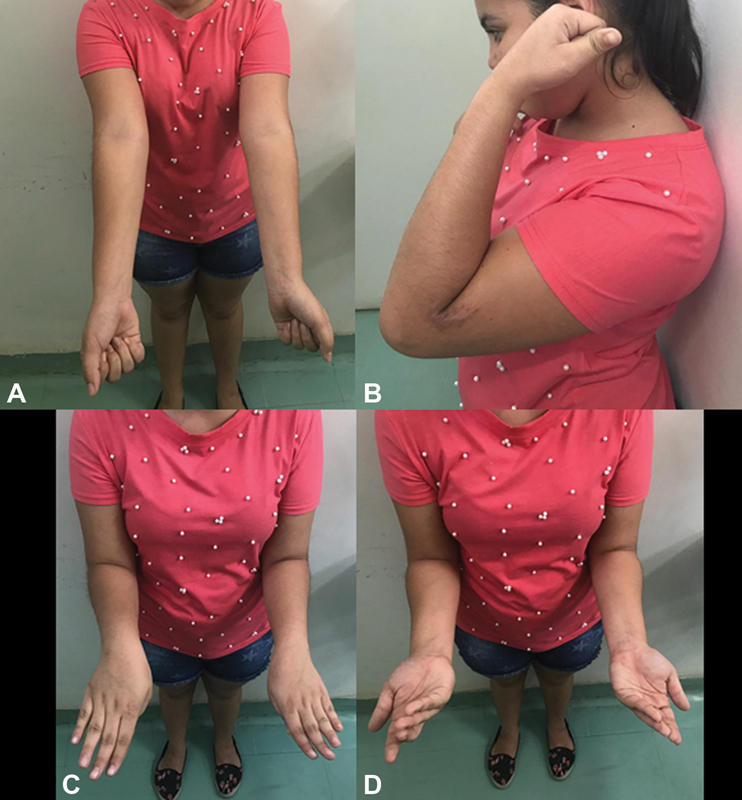
At 12 months postoperatively, the patient had symmetric range of motion, with no residual instability.

**Fig. 5:**
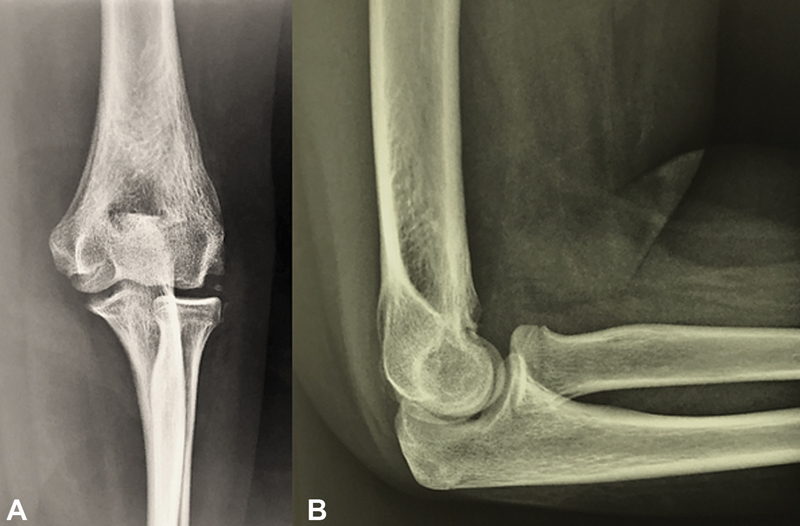
Lateral ( **A** ) and AP ( **B** ) radiographs of the elbow 12 months after surgery, showing no evidence of recurrence of HO.

The patient was submitted to general anesthesia and brachial plexus block. A direct lateral approach was performed, followed by excision of the HO by means of osteotomy and osteoplasty ( Fig. 3 ). A complete ROM was achieved. The day after surgery, she was advised to perform active and passive kinesiotherapy, progressing to physical rehabilitation after seven days. No additional prophylaxis for HO was administered. At 12 months postoperatively, the patient had pain-free symmetric ROM, with no residual instability, preserved strength, and radiographs with no evidence of recurrence of the HO ( Figs. 4 and 5 ).

## Discussion

Dislocation of the elbow joint is an uncommon injury in younger children, who usually experience elbow dislocation in association with fractures, particularly of the medial epicondyle, proximal radius, and coronoid process. 1 2 3 Pediatric elbow dislocations with no concomitant fractures are rare, with few cases reported in the literature. 1 3 6 7 8 The complications of elbow dislocation are mostly related to neurologic (10%) and vascular injuries (6% to 8%), HO and loss of motion, recurrent dislocations, radioulnar synostosis, and cubitus recurvatum. 1 6 7 8 9

Posttraumatic insult is the most common cause of HO, typically following fractures, dislocations, and operative procedures, accounting for up to 75% of the cases. 5 7 9 However, it is uncommonly symptomatic, especially in the pediatric age group. 5 6 8 9 In this scenario, the damage to the brachialis muscle fibers and the formation of a focal hematoma may predispose to the development of the HO. 5 9 Furthermore, a recent study 5 suggested that overweight and obesity in pediatric patients may be a risk factor for HO. In the case herein presented, the child was overweight, a factor that may have contributed to the occurrence of HO.

Susnjar et al. 8 reported a case of a 9-year-old girl with formation of HO at the elbow after the surgical treatment of a fracture of the lateral humeral condyle. The HO was surgically removed eight months after the first surgery. Araoojis et al. 6 reported a unique case of complete medial elbow dislocation in a 10-year-old boy who underwent closed reduction under general anesthesia. After 2 years of follow-up, the radiograph showed matured HO along the anterior capsule, but the patient had full elbow ROM, and did not require surgery.

In the literature, the prophylaxis and drug prevention for HO are controversial. Drug prophylaxis with indomethacin and other nonsteroidal anti-inflammatory drugs have been advocated in the early stages and after surgical excision. 4 In contrast, indomethacin has been reported as a non-effective prophylaxis for HO after surgery for acetabular fractures. 10 Radiotherapy has also been suggested as effective to prevent HO if performed 24 hours preoperatively or up to 72 hours postoperatively. 4 Nevertheless, evidence for the prophylaxis with indomethacin or irradiation to prevent posttraumatic HO is lacking for the pediatric population. 1 6 8

We described a unique case of a child who sustained a complete elbow dislocation and partial disruption of the brachialis muscle, evolving with HO and loss of ROM. The patient underwent surgical treatment and resection of the HO with an excellent result, with no recurrence. We recommend the maturation of the HO to program a surgical treatment, and we did not administer prophylaxis for HO beyond early kinesiotherapy.
