# Supplementary material for: Heterotopic Ossification Following Pediatric Elbow Dislocation: A Case Report
Source: Rev Bras Ortop (Sao Paulo). 2021 Dec 16;59(Suppl 2):e184–7. doi: 10.1055/s-0041-1739403 (PMC11679626; doi:10.1055/s-0041-1739403)
Supplement: Related texts

# Research article: Ossificação heterotópica após luxação pediátrica do cotovelo: Um relato de caso

**Keywords:** adolescente, articulação do cotovelo, criança, luxações articulares, ossificação heterotópica

## Abstract

A luxação do cotovelo é relativamente incomum em crianças, sendo que a maioria dos casos ocorre com fraturas associadas. A luxação isolada do cotovelo é relativamente rara, podendo implicar em uma maior probabilidade de lesões graves dos tecidos moles. Uma possível complicação é o desenvolvimento da ossificação heterotópica, que geralmente é assintomática, mas pode alterar o movimento articular. Descrevemos o caso de uma menina de 11 anos que sofreu luxação do cotovelo sem fraturas associadas, mas com ruptura parcial distal do músculo braquial. Após a redução fechada, a paciente desenvolveu ossificação heterotópica no terço distal anterior do úmero, com perda da amplitude do movimento articular do cotovelo. O tratamento cirúrgico, com excisão da ossificação heterotópica, por meio de uma abordagem lateral direta, proporcionou um excelente resultado. A ossificação heterotópica é uma possível complicação após luxação do cotovelo em crianças, sendo que a excisão cirúrgica mediante uma abordagem lateral é sempre uma opção de tratamento quando há limitação funcional. Antes de ser tomada a decisão cirúrgica, a maturação do processo de ossificação deve ser observada.

## Introdução

O cotovelo é a articulação que mais comumente apresenta luxação traumática na infância; embora a lesão seja relativamente rara, representa entre 3% e 25% de todas as lesões pediátricas do cotovelo. 1 As luxações pediátricas do cotovelo são lesões complexas, associadas a fraturas em 75% dos casos, ao passo que as luxações simples (puramente ligamentares) em crianças estão associadas a vários graus de lesões dos tecidos moles. 1 2

Após luxação do cotovelo, os pacientes podem apresentar perda variável da amplitude de movimento (ADM) associada ou não à ossificação heterotópica (OH). 3 A OH é o desenvolvimento do osso lamelar maduro dentro de tecidos além do periósteo, como músculo esquelético, componentes fibrosos, capsuloligamentares, e tecido subcutâneo. 4 Na maioria dos casos, a OH é assintomática, podendo ser detectada como um achado de imagem incidental. No entanto, pode ser dolorosa e estar associada a disestesia focal, sinais inflamatórios, e redução da ADM. A OH sintomática na região do cotovelo em crianças é um achado incomum, especialmente sem uma fratura associada ou histórico de cirurgia. 4 5 Aqui, nós descrevemos o caso de uma menina de 11 anos com OH após uma luxação traumática do cotovelo sem fraturas associadas, mas com ruptura parcial do músculo braquial. Durante o acompanhamento, a paciente apresentou perda persistente da flexão, sendo submetida a excisão cirúrgica da OH, que resultou na normalização do movimento do cotovelo, e não houve recorrência.

## Relato de caso

Este estudo foi aprovado pelo Comitê de Ética em Pesquisa (sob o protocolo n° 36203120.6.0000.0023), com a anuência da participante e de seus pais.

Uma menina de 11 anos deu entrada no pronto-socorro relatando queda de pequena altura, com trauma indireto no cotovelo esquerdo. Não havia histórico de lesão musculoesquelética no braço acometido. Durante o exame físico, apresentou dor intensa, edema, sensibilidade, deformidade do cotovelo, sem déficit neurovascular. As radiografias mostraram luxação posterolateral do cotovelo sem fraturas concomitantes. Foi realizada a redução fechada sob anestesia, e imobilizada com tala gessada posterior com 90o de flexão e rotação neutra do cotovelo durante uma semana, seguida de duas semanas com tipoia de ombro. Porém, devido a edema e equimose importantes do cotovelo, foi realizada uma ressonância magnética (RM), que evidenciou ruptura parcial das fibras do músculo braquial e acúmulo de sangue ( Fig. 1 ). Nenhum tratamento específico foi realizado para tratar a lesão do músculo braquial. Após seis meses de fisioterapia, a paciente apresentou ADM indolor, com aproximadamente 90° (flexão de 95° e déficit de extensão de 5°). Não foi observada nenhuma perda nos movimentos de pronação e supinação. Uma massa sólida considerável era palpável na extremidade distal do úmero. As radiografias e a tomografia computadorizada do cotovelo mostravam OH na face anterior do úmero distal, provocando potencial impacto articular ( Fig. 2 ). Por causa da perda residual do ADM, decidiu-se ressecar a OH cirurgicamente.

**Fig. 1:** ( **A** ) Radiografia de menina de 11 anos de idade com incidências de perfil ( **B** ) e anteroposterior (AP), mostrando luxação posterolateral do cotovelo sem fraturas concomitantes; ( **C** ) radiografia com incidência de perfil e ( **D** ) AP, mostrando articulação congruente sem fraturas, após redução fechada. ( **E** ) Ressonância magnética: corte sagital ponderado em T2 do cotovelo esquerdo, após a luxação traumática e a redução fechada. A ressonância magnética mostra ruptura parcial das fibras do músculo braquial e acúmulo de sangue de 3,1 cm × 1,8 cm com baixa resolução.

**Fig. 2:** ( **A** ) Imagem radiográfica em perfil, ( **B** ) tomografia computadorizada em corte sagital, ( **C** ) corte axial, e ( **D** ) em 3D do cotovelo, mostrando ossificação heterotópica (OH) na face anterior do úmero distal, provocando potencial impacto articular.

A paciente recebeu anestesia geral e bloqueio do plexo braquial. Foi realizada abordagem lateral direta, seguida de excisão da OH, por meio de osteotomia e osteoplastia ( Fig. 3 ). Foi restaurado o ADM completo após a excisão da OH. No dia seguinte à cirurgia, a paciente foi orientada a realizar cinesioterapia ativa e passiva, iniciando a reabilitação física após sete dias. Nenhuma profilaxia adicional foi administrada para a OH. Aos 12 meses do pós-operatório, a paciente apresentava ADM simétrica, indolor, sem instabilidade residual, força preservada, e radiografias sem evidência de recorrência da OH ( Figs. 4 e 5 ).

**Fig. 3:** ( **A** ) Imagem do intraoperatório mostrando a ossificação heterotópica do úmero distal por meio da abordagem lateral direta. ( **B** ) Visualização radiológica intraoperatória em perfil do cotovelo, após a excisão da OH.

**Fig. 4:** Aos 12 meses de pós-operatório, a paciente apresentava amplitude de movimento simétrica, sem instabilidade residual.

**Fig. 5:** ( **A** ) Radiografias em AP ( **A** ) e perfil ( **B** ) do cotovelo 12 meses após a cirurgia, sem evidências de recorrência de OH.

## Discussão

A luxação da articulação do cotovelo é uma lesão incomum em crianças mais novas, que geralmente apresentam luxação do cotovelo em associação a fraturas, particularmente do epicôndilo medial, do rádio proximal e do processo coronoide. 1 2 3 As luxações pediátricas do cotovelo sem fraturas concomitantes são raras; há poucos casos relatados na literatura. 1 3 6 7 8 As complicações da luxação do cotovelo estão principalmente relacionadas a lesões neurológicas (10%) e vasculares (6% a 8%), OH, perda de movimento, luxações recorrentes, sinostose radioulnar, e *cubitus recurvatum* . 1 6 7 8 9

As lesões pós-traumáticas são as causas mais comuns da OH, geralmente após fraturas, luxações e procedimentos cirúrgicos, sendo responsáveis por até 75% dos casos. 5 7 9 No entanto, não é comum que a OH apresente sintomas, especialmente na faixa etária pediátrica. 5 6 8 9 Nesse cenário, o dano às fibras do músculo braquial e a formação de um hematoma focal podem predispor ao desenvolvimento da OH. 5 9 Além disso, um estudo recente 5 sugeriu que o sobrepeso e a obesidade em pacientes pediátricos podem ser fatores de risco para a OH. No caso apresentado, a criança apresentava sobrepeso, fator que pode ter contribuído para a ocorrência da OH.

Susnjar et al. 8 relataram o caso de uma menina de 9 anos, com formação de OH no cotovelo, após o tratamento cirúrgico de uma fratura do côndilo lateral do úmero. A OH foi removida cirurgicamente oito meses após a primeira cirurgia. Araoojis et al. 6 relataram caso único de luxação medial total do cotovelo em um menino de 10 anos submetido a redução fechada sob anestesia geral. Após acompanhamento de 2 anos, o exame radiográfico mostrou OH amadurecida, junto com a cápsula anterior, mas o paciente apresentava ADM completo do cotovelo, e não houve necessidade de tratamento cirúrgico.

Na literatura, a profilaxia e a prevenção medicamentosa para a OH são controversas. A profilaxia medicamentosa com indometacina ou outros anti-inflamatórios não esteroides tem sido defendida nos estágios iniciais e após a excisão cirúrgica. 4 Em contraste, a indometacina foi relatada como profilaxia não eficaz para OH após cirurgia para fraturas acetabulares. 10 A radioterapia também tem sido sugerida como eficaz para prevenir a OH, caso seja realizada nas primeiras 24 horas do período pré-operatório, ou até 72 horas no pós-operatório. 4 No entanto, a população pediátrica carece de evidências profiláticas relacionadas à indometacina ou à radioterapia com o objetivo de prevenir a OH pós-traumática. 1 6 8

Descrevemos o caso único de uma criança que apresentou luxação do cotovelo e ruptura parcial do músculo braquial, e evoluiu com OH e perda do ADM. A paciente foi submetida a tratamento cirúrgico e ressecção da OH com excelentes resultados, sem recidiva. Recomendamos aguardar a maturação da OH, a fim de que um tratamento cirúrgico possa ser programado, não administrando profilaxia para a OH além da cinesioterapia precoce.
